# Factors affecting the identification of individual mountain bongo antelope

**DOI:** 10.7717/peerj.1303

**Published:** 2015-11-12

**Authors:** Gwili E.M. Gibbon, Markus Bindemann, David L. Roberts

**Affiliations:** 1 Durrell Institute of Conservation & Ecology, School of Anthropology & Conservation, University of Kent, Canterbury, Kent, UK; 2 School of Psychology, University of Kent, Canterbury, Kent, UK

**Keywords:** Individual recognition, Camera trap, Observer error, Markings, Photographs, *Tragelaphus eurycerus isaaci*

## Abstract

The recognition of individuals forms the basis of many endangered species monitoring protocols. This process typically relies on manual recognition techniques. This study aimed to calculate a measure of the error rates inherent within the manual technique and also sought to identify visual traits that aid identification, using the critically endangered mountain bongo, *Tragelaphus eurycerus isaaci*, as a model system. Identification accuracy was assessed with a matching task that required same/different decisions to side-by-side pairings of individual bongos. Error rates were lowest when only the flanks of bongos were shown, suggesting that the inclusion of other visual traits confounded accuracy. Accuracy was also higher for photographs of captive animals than camera-trap images, and in observers experienced in working with mountain bongos, than those unfamiliar with the sub-species. These results suggest that the removal of non-essential morphological traits from photographs of bongos, the use of high-quality images, and relevant expertise all help increase identification accuracy. Finally, given the rise in automated identification and the use of citizen science, something our results would suggest is applicable within the context of the mountain bongo, this study provides a framework for assessing their accuracy in individual as well as species identification.

## Introduction

Camera-traps are integral to the monitoring of many otherwise cryptic species. First used to study wildlife in the early 20th century (Chapman, 1927), modern technological advances have facilitated the production of affordable units that can operate for months in extreme conditions (O’Connell, Nichols & Karanth, 2011). As a result, camera traps have become increasingly popular and are employed by numerous conservation projects (Rowcliffe & Carbone, 2008), providing key ecological and behavioural information that would otherwise be difficult to gather (O’Connell, Nichols & Karanth, 2011).

The use of camera-traps has been particularly prevalent within studies monitoring highly mobile forest species, such as large and rare felids (Carbone et al., 2001). Notable cases include tigers, *Panthera tigris*, where they provided data integral to creating the first accurate estimates of population densities and population size (Karanth, 1995; Karanth & Nichols, 1998), and the jaguar, *P. onca*, which has been the focus of at least 83 different camera-trap survey efforts (O’Connell, Nichols & Karanth, 2011). Such monitoring techniques are often reliant on manual visual identification, by human observers, of individual animals from the target species. This approach is fundamental in creating estimates of population density and size, using processes such as mark-capture-recapture (Karanth, 1995; Paviolo et al., 2008). Conservation scientists also employ such manual identification techniques to monitor the movement and survivorship of individuals of a species (Graham & Roberts, 2007). This application has been particularly prevalent in studies of pelagic species, such as the whale shark, *Rhincodon typus* (Arzoumanian, Holmberg & Norman, 2005; Bonfil et al., 2005; Graham & Roberts, 2007; Jorgensen et al., 2010) and baleen whales (Katona et al., 1979; Katona & Whitehead, 1981; Fujiwara & Caswell, 2002a; Fujiwara & Caswell, 2002b).

While the manual identification technique has the ability to provide a wealth of data, it is also prone to observer bias and highly labour-intensive. These techniques are, for example, typically reliant on the management of large image libraries and manual comparisons can require 30–40 minutes per image (Hiby et al., 2009). In an attempt to reduce the workload of species identification, some projects therefore compromise on expertise by using ‘citizen scientists’ to identify individuals for them (Silvertown, 2009). These are non-professional observers with a personal interest in conservation, who volunteer to help with species monitoring.

The use of citizen scientists has brought into focus the question of the extent to which identification errors might occur during species monitoring by such non-professionals and, more generally, of the error rates that might be inherent within the manual technique (e.g., Morrison et al., 2011). This problem is compounded because these techniques rely on a variety of visual traits, depending on the species under observation. For example, some techniques utilise a species’ unique coat or skin patterning, such as for tigers (Hiby et al., 2009), plains zebra *Equus quagga* (Lahiri et al., 2011), bobcats *Lynx rufus* (Mendoza et al., 2011), and the narwhal *Monodon monocerus* (Auger-Méthé, Marcoux & Whitehead, 2011). Another approach is to identify individuals through the use of other visual traits, such as iris patterning (Rocha, Carrilho & Rebelo, 2013), whisker spots (Anderson et al., 2010), and implanted elastomers (Bendik et al., 2013). Indigenous communities have also used footprints for millennia (Stander, 1997), and these techniques have been adapted for use by non-expert trackers and conservation biologists (Sharma, Jhala & Sawarkar, 2005; Alibhai, Jewell & Law, 2008; Gu et al., 2014).

In light of the increasing use of camera traps for species monitoring, the emergence of citizen science, and the variety of traits that can be used for individuation, this study sought to begin to explore how these factors affect the accuracy of manual species identification. For this purpose, we focus on the eastern or mountain bongo (*Tragelaphus eurycerus isaaci*). This is a critically endangered subspecies of antelope endemic to the montane forests of Kenya (Kingdon, 1982). The mountain bongo is the focus of an international restoration effort (Reillo, 2004; Veasey, 2010) and is utilised as a ‘flagship species’ (Caro, 2010) for the conservation of Kenya’s montane forests (CBSG, 2010). These highland forests are recognised as a part of Kenya’s natural heritage and provide valuable ecosystem services that are critical to the health of the nation’s environmental and economic systems (Akotsi & Gachanja, 2004).

In the 1960s, the global population of mountain bongo was estimated at 1,000 individuals, but by the turn of the 21st century many populations had become extinct (e.g., Cherengani Hills and Chupungulu forests). Currently, the *in-situ* population is estimated at 100–150 individuals, which exist in seven populations within three highland complexes; the Aberdares range (60–80 individuals in three potentially discrete populations), Mount Kenya (∼10 individuals) and the Mau/Eburru forest complex (∼30 individuals in 3 discrete populations). In contrast, over 700 mountain bongo exist in captivity (Bosley, 2011). The main drivers of decline are hypothesised to be predominantly anthropogenic, with habitat loss, overhunting and epizootic events from diseases, such as rinderpest, identified as the chief causes (Prettejohn, 2008; Estes et al., 2011).

Mountain bongo are predominantly nocturnal, extremely shy and, due in part to excessive hunting pressure, survive only in remote and inaccessible areas. In these areas, camera-traps have proven to be the most effective way to gather data with which to monitor populations and create estimates of population size. Such techniques rely on the labour-intensive process of manual visual identification of individuals by experts. This is possible due to the wide degree of polymorphism in horn shape, coat colouration and patterning, which is reported to be used for individual identification by zookeepers (G Gibbon, pers. obs., 2014). Empirical observations from camera-trap data of wild populations have also found mountain bongo to exhibit distinctive coat pattering, with individual differences in the stripe on each flank being a chief trait to aid in the identification of individuals (M Prettejohn, pers. comm., 2014). Those working in mountain bongo conservation have highlighted a need to further understand the validity of selecting such visual traits for this purpose.

To begin to address these questions, the current study explored observers’ identification accuracy of individual mountain bongo. Specifically, we sought to investigate several key factors, comprising identification accuracy for (a) different visual traits (hereafter ‘image trait type’); of (b) camera-trap photographs versus images of captive individuals (hereafter ‘image source’); and of (c) expert versus non-expert observers (hereafter ‘expert level’). For this purpose, we employed a two-alternative forced-choice (2AFC) paradigm, in which observers had to decide whether pairs of photographs depict the same or two different individuals of a species. This paradigm has been used extensively in other domains, such as the forensic identification of human faces (Johnston & Bindemann, 2013). In the study of face identification, this task is held to provide a highly optimized scenario that can be used to establish the upper limits of possible performance (Burton, White & McNeill, 2010). It has also been used to assess the contribution of different features (i.e., ‘traits’) (Estudillo & Bindemann, 2014; Megreya & Bindemann, 2009) and to compare professionals in facial identification, such as passport officers, with non-professional observers (White et al., 2014). This paradigm therefore seems well-suited to establish the accuracy of professional and non-professional observers in the identification of mountain bongo antelope from different traits and image types.

## Materials and Methods

This study was conducted as a survey that was disseminated through emails and social media using the online survey platform SurveyGizmo™ (http://www.surveygizmo.com/) to two main groups of participants. The first was a general interest group of students and staff within the School of Anthropology and Conservation, University of Kent, who had no prior experience of bongo identification. The second group comprised experts in the identification of bongo antelopes, including members of the Bongo Surveillance Project (BSP), Kenya Wildlife Service (KWS), and members of the European Association of Zoos and Aquaria Eastern Bongo Endangered Species Programme. The survey ran for four weeks, starting on the 3rd April 2014.

The survey was divided into three stages. The first introduced the study and informed consent was obtained; the second recorded basic demographic information and participants’ experience in working with mountain bongos; the third stage consisted of the matching task and comprised 84 side-by-side pairs of mountain bongo images in a random order. Participants were asked to decide whether each pair depicted the same bongo or two different individuals.

**Figure 1:**
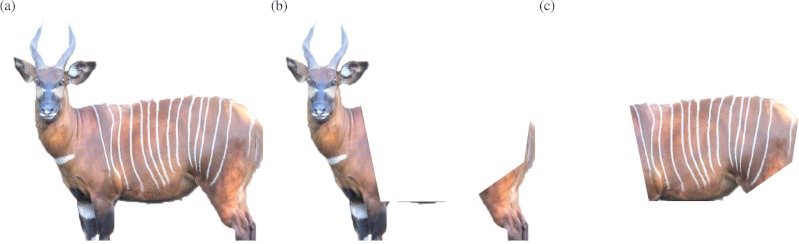
Examples of the three trait types. (A) Side profile ‘full side’; (B) side profile with the flank removed ‘no flank’ and (C) ‘flank only’.

A library of images was constructed from a number of different sources. The Bongo Surveillance Project (BSP) provided camera-trap images, while images of captive individuals were obtained from breeding institutions through the Eastern Bongo Studbook (Bosley, 2011). Additional photographs, taken by the primary author (GEMG), were then used to supplement these. Suitable images were selected from the library based on the angle of the individual; only those where the individual was less than 20° off parallel to the anteroposterior axis and with a body orientation that clearly showed the head, horns and flank. Owing to their greater representation in captivity and the species’ sexual dimorphism, only images of mature females were used. A total of 84 images was selected; including 24 camera-trap images of five individuals and 60 images from 22 captive individuals. These images were then edited, using Adobe Photoshop CS6™ to remove the background, the dorsal crest and the legs below the ankle joint to eliminate background information that could provide image context and therefore bias responses. Three image trait types were created for each image (Fig. 1), comprising (A) the complete side profile (‘full side’—unaltered except for the removal of the background, dorsal crest and ankle), (B) the side profile with stripes on the flank removed (‘no flank’), and (C) the removed section of the flank (‘flank only’).

**Figure 2:**
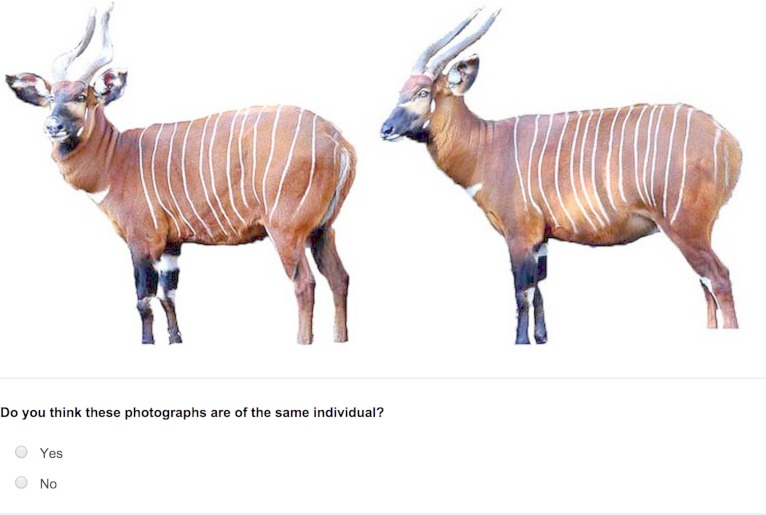
An example of a mismatch side-by-side pairing.

For each of these image trait types, two types of stimulus pairs were constructed. One type depicted identity ‘matched pairings’, and consisted of two different images of the same individual. The other were ‘mismatched pairings’ and comprised photographs of two different individuals (Fig. 2). The identities for these ‘mismatched pairings’ were selected using a random number generator. Based on this process, four pairings for each side and for each image trait type were constructed from the camera-trap images producing 24 pairings. This process was repeated for the images of captive individuals, with 10 pairings constructed for each side and for each of the three image trait types, producing 60 pairings. Overall, this resulted in a total of 84 side-by-side stimulus pairings.

## Results

Data were downloaded from SurveyGizmo. A total of 456 responses were received. Of these, 227 (49.8%) were complete and used for analysis. Thirty-five respondents (15.4%) indicated that they worked with mountain bongo and were deemed ‘experts’; the remaining 192 respondents (84.6%), who did not work with mountain bongo, were deemed ‘non-experts’.

The mean percentage of correct responses was then calculated for these participants as a function of image trait type (full side, no flank, flank only), expertise (experts vs. non-experts), and pairing type (matched vs. mismatched pairings). To ensure a normal distribution of the percentage data, this was transformed using an arcsine-square root transformation and entered into SPSS 21™ (IBM Corp, 2012), which was used for the statistical analyses.

### Accuracy across image trait type

**Figure 3:**
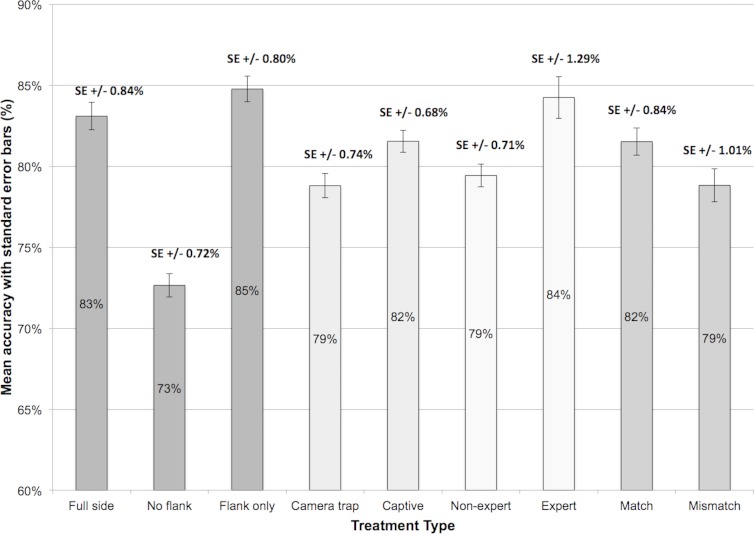
Mean accuracy and standard error (%) across the nine treatment types within the four factors.

Overall, the mean percentage of correct responses was 80.1% (±14.0 SE). The percentage accuracy for individual factors is displayed in Fig. 3. For image trait type, this data shows that accuracy was highest in the ‘flank only’ (1.20 mean arcsine % correct, ±0.01 SE) and ‘full side’ conditions (1.18 mean arcsine % correct, ±0.01 SE), and lowest for ‘no flank’ displays (1.03 mean arcsine % correct, ±0.01 SE). In line with these observations, a one-way ANOVA revealed an effect of image trait type, *F*(2, 678) = 74.87, *p* < 0.001. Tukey post-hoc test showed that accuracy was comparable for ‘full side’ and ‘flank only’ displays (*p* = 0.23), but was lower in the ‘no flank’ condition compared to ‘full side’ (*p* < 0.001) and ‘flank only’ (*p* < 0.001) image pairs.

### Accuracy for different image sources

Image source also affected performance in bongo identification (see Fig. 3), with lower accuracy for camera-trap images (1.11 mean arcsine % correct, ±0.01 SE) than photographs of captive individuals (1.14 mean arcsine % correct, ±0.01 SE), *t*(226) = 4.22, *p* < 0.001.

### Accuracy for pairing type

Accuracy was higher for match (1.15 mean arcsine % correct, ±0.01 SE) than mismatch image pairings (1.12 mean arcsine % correct, ±0.01 SE) but this difference was not reliable, *t*(226) = 1.81, *p* = 0.07.

### Accuracy of expert and non-expert observers

Expert observers, with professional experience working with bongo antelope, achieved higher identification accuracy (1.17 mean arcsine % correct, ±0.02 SE) than non-experts (1.11 mean arcsine % correct, ±0.01 SE), *t*(225) = 2.87, *p* < 0.01. In addition, three two-way ANOVAs were conducted to investigate the cross-factor relationship between expert level and the other factors. No interaction was found between expert level and image trait type, *F*(2, 450) = 1.17, *p* = 0.31, image source, *F*(1, 225) = 2.92, *p* = 0.09, or pairing type, *F*(1, 225) = 0.71, *p* = 0.40.

## Discussion

This study highlights how specific morphological traits vary in their usefulness when humans are identifying individual mountain bongo. The flank, featuring only the abdominal stripes, yielded the highest accuracy rate, followed by the full side profile of the entire individual. By contrast, removal of the flank reduced identification accuracy. This suggests that stripes on the flank are the most informative trait here, and are key to maximising identification accuracy. This finding converges with suggestions that many species exhibit unique coat patterns that aid the identification of individuals (Hiby et al., 2009).

The comparison of images of individuals from camera-traps and captive images was also undertaken to give a more realistic application. The fact that accuracy was significantly, although numerically only marginally, lower for camera-trap images may be unsurprising, given that these were of lower quality and often captured at night. Thus, those involved in camera-trapping should strive to use cameras of the highest quality to reduced the inherent error rate, although this may need to be weighed against the higher costs of doing so.

The identification accuracy of non-experts and experts is perhaps more surprising. While non-experts’ accuracy was compromised considerably, with one in five responses reflecting an identification error (79% correct), experts performed better (84% correct). This indicates that experience with the species may have, consciously or unconsciously, trained these people to pick up on subtle differences in morphology and select traits that aid in identification. Generally, however, the difference in accuracy between experts and non-experts was numerically also relatively small (∼5%). This indicates that non-experts, such as citizen scientist, could be used for species monitoring without compromising this process dramatically.

However, it is also notable that experts’ performance was far from perfect, with an identification error still being made on one in every six trials. This was observed with an optimized matching task, in which respondents have a 50:50 chance of answering correctly. In other domains, such as forensic face identification, this level of matching performance would be considered problematic and raise concern about identification accuracy in applied settings (Johnston & Bindemann, 2013; White et al., 2014). The current data should raise similar questions about the accuracy of manual species monitoring in the field, by both experts and non-experts.

We draw this conclusion with some caveats. It is possible, for example, that our method also inflated errors by not giving participants an ‘opt out’ option when they were unsure of the correct answer. While this might force participants to make identification guesses on some trials, research on the consistency of face matching suggests that such guesses tend to inflate, rather than decrease, accuracy (Bindemann, Avetisyan & Rakow, 2012). Ultimately, however, this issue clearly demands further investigation.

We also have no data on the training that our experts had previously received in bongo identification. Accuracy should improve in non-experts, such as citizen scientists, with similar training. However, it might also vary in both non-expert and expert observers with different training protocols. To this point, we note also that expertise did not interact with image trait type, image source and pairing type. This suggests that these aspects of bongo identification might be determined by factors other than training and expertise.

## Conclusions

This study and its findings provide a valuable methodology and important recommendations for those involved in monitoring *in-situ* mountain bongo populations, as well as other species with features that may help discriminate between individuals. We have shown that the mean human ability to accurately distinguish between individual mountain bongo is at 79% and 84% in non-expert and expert observers under highly optimized conditions. In turn, this indicates that a considerable number of errors might occur during species monitoring using manual identification techniques.

We have also shown that the selection of only the stripes on the flank increases identification accuracy, whereas the inclusion of other traits can confound visual discriminatory ability, and that identification accuracy is lower for camera-trap images. To improve identification accuracy in the field, monitoring efforts should therefore strive to use the highest quality camera-traps possible and place at least some of these perpendicular to game trails so as to capture images of the flank of individuals as they visit mineral licks or waterholes. Our findings also bear relevance to those aiming to develop automated individual identification systems, suggesting that they could focus solely on the stripes on the flank as, at least within a human-based identification system, other features seem to confound identification.

We recognise that the error rates in this study might reflect the within-subject variation in our stimulus set. It is well known, for example, that camera-trap images are characterised by a wide range of body postures. In future, it may be possible to reduce this variation by developing three-dimensional models that have the ability to translate a 3D image onto a flat surface. This approach has already allowed seized tiger skins to be matched to images of once-living tigers and such models are important for reducing misidentification (Hiby et al., 2009). For the mountain bongo, we would also recommend the establishment of a central database of *in-situ* images. This would be an important tool for population monitoring, but also for the development of an automated individual identification system. Such systems, or ‘zoometrics’, are currently a rapidly growing field in conservation (Hoque, Azhar & Deravi, 2011). Our findings suggest that, for the identification of mountain bongo, the development of such systems should focus specifically on the flank region. As such, our data support current efforts in the development of automated individual identification systems that already focus on the flank regions of other species, such as tigers and zebras (e.g., Hiby et al., 2009; Lahiri et al., 2011). The current data also provide a baseline of human performance against which the identification accuracy of such automatic systems could be compared.

## Supplemental Information

10.7717/peerj.1303/supp-1 Supplemental Information 1 G Gibbon et al. full dataset
